# Biological characteristics of a sub-population of cancer stem cells from two triple-negative breast tumour cell lines

**DOI:** 10.1016/j.heliyon.2021.e07273

**Published:** 2021-06-10

**Authors:** Javier Enciso-Benavides, Luis Alfaro, Carlos Castañeda-Altamirano, Nancy Rojas, José González-Cabeza, Nathaly Enciso, Fernando Riesco, Miluska Castillo, Javier Enciso

**Affiliations:** a Grupo de Medicina Regenerativa, Universidad Científica del Sur, Lima, Peru; b Laboratorio de Células Madre, Clínica Veterinaria Enciso, Lima, Peru; c Laboratorio de Microscopia Electrónica, Instituto de Patología, UNMSM, Lima, Peru; d Instituto Nacional de Enfermedades Neoplásicas, Lima, Peru; e Universidad Privada Antenor Orrego, Trujillo, Peru; f Dirección General de Investigación, Desarrollo e Innovación, Universidad Científica del Sur, Lima, Peru

**Keywords:** Triple-negative breast tumors, CD44^*high*^/CD24^*low*^ phenotype cells, Cancer stem cells, Magnetic immunoselection

## Abstract

Triple-negative breast tumours (TNBTs) make up 15–20% of all breast tumours. There is no treatment for them, and the role that cancer stem cells (CSCs) have in carcinogenesis is still unclear, so finding markers and therapeutic targets in CSC exosomes requires these cells to exist as a homogeneous cell population. The objective of this work was to determine differences in ultrastructural morphology, proliferative capacity, and mouse-xenotransplantation characteristics of the MDA-MB-231 and MDA-MB-436 TNBT cell lines with the CD44^*high*^/CD24^*low*^ phenotype in order to study their exosomes. The results show that the CD44^*high*^/CD24^*low*^ MBA-MB-231 cells had a population doubling time of 41.56 h, compared to 44.79 h in the MDA-MB-436 cell line. After magnetic immunoseparation, 18.75% and 14.56% of the stem cell population of the MDA-MB-231 and MDA-MB-436 cell lines, respectively, were of the CD44^*high*^/CD24^*low*^ phenotype, which were expanded to reach purities of 80.4% and 87.6%. The same expanded lineage in both cell lines was shown to possess the pluripotency markers Nanog and Oct4. Under a scanning electron microscope, the CD44^*high*^/CD24^*low*^ lineage of the MBA-MD-231 cell line formed groups of more interconnected cells than this lineage of the MBA-MD-436 line. A total of 16% of the mice inoculated with the CD44^*high*^/CD24^*low*^ lineage of either cell line presented tumours of the breast, lung, and submandibular ganglia, in whose tissues variable numbers of inoculated cells were found 30 days post-inoculation. By magnetic immunoselection, it was possible to isolate in similar quantities and characterize, expand, and xenotransplant the CD44^*high*^/CD24^*low*^ lineage of the MDA-MB-231 and MDA-MB-436 cell lines. The former cell line has greater proliferative capacity, the two lines differ under scanning electron microscopy in how they intercommunicate, and both cell lines induce new tumours in mice and persist at least 30 days post-inoculation in the transplanted animal so their exosomes would also be different.

## 1 Introduction

Breast cancer includes very heterogeneous tumours, and a wide spectrum of factors are responsible for variations in therapeutic response and prognosis [1]. Worldwide, breast cancer represents 11.6% of all cancers [2], of which 15–17% are triple-negative breast tumours (TNBT) [3]. The latter are a heterogeneous group of tumours characterized by aggressive behaviour, high risk of metastasis, and poor survival, and they comprise several biologically distinct subtypes [4, 5]. A better understanding of this biological heterogeneity will allow more effective and individualized treatment approaches [6].

As part of this effort, many researchers have focussed on cancer stem cells (CSCs), which have a greater tolerance to chemotherapy, hormonal therapy, and radiotherapy and are capable of reproducing the majority of the tumour after killing the populations of cells sensitive to first-line therapy, leading to relapse of the disease [7]. To identify these cancer stem cells, several new cell-surface markers of breast cancer associated with the CSC lineage have been discovered, such as the presence of CD44 (CD44^*high*^) combined with the (near-)absence of CD24 (CD24^*low*^), so CD44 was deemed an important biomarker and therapeutic target of this cancer [8].

The expression of markers of CSC populations varies between subtypes of breast cancer and cancers with different clinicopathological characteristics. Thus, each CSC population can have a different clinical importance in different phenotypes of breast cancer [9], but it is accepted that they are potential key players that drive the aggressiveness of TNBTs [10]. This heterogeneity of CSCs has led to the search for a therapeutic strategy based on the combination of multiple targets [11].

The consensus markers for breast cancer stem cells are CD44^*high*^/CD24^*low*^. Two hundred CSCs with CD44^*high*^/CD24^*low*^ phenotype could initiate tumours [12] and were important for resistance to chemotherapy and for tumour metastasis [13]. Other authors suggested that the CD44^*high*^/CD24^*low*^ phenotype that is associated with invasion is not sufficient for the establishment of lung metastasis from some types of tumour [14], but recent studies show that these CD44^+^/CD24^-^ cells show an epithelial mesenchymal transition (EMT) phenotype with a high tumourigenic capacity for invasion and metastasis [15].

Other immunophenotypes obtained by immunoselection, CD44^*high*^/CD24^*high*^*,* CD44^*low*^/CD24^*high*^, and CD44^low^/CD24^low^, have been described, which show in vitro morphological and population size differences under immunomagnetic separation, which could be related to pathogenic differences between these immunophenotypes described that have been in other studies [16]. Thus, the CD24 phenotype is related to susceptibility and resistance to chemotherapeutic agents in vivo, while in patients with TNBTs, it is implicated in resistance to docetaxel and doxorubicin. Other phenotypes, such as CD44^+^/CD24^+^, are more resistant to docetaxel, while the CD44^*high*^/CD24^*low*^ phenotype is resistant to doxorubicin [17]. Very early studies found that when evaluating 13 breast cancer cell lines, five of them (MDA-MB-231, MDA-MB-436, Hs578T, SUM1315, and HBL-100) had a high percentage (˃ 30%) of the CD44^*high*^/CD24^*low*^ immunophenotype with basal/mesenchymal markers or myoepithelial markers but no luminal markers, as well as high levels of expression of the pro-invasive proteins IL-1a, IL-6, IL-8, and urokinase-type plasminogen activator (UPA). Of these five cell lines, the MDA-MB-231 cell line is the only one that has the property of expressing a wide range of genes that favour bone and lung metastasis [14].

Regarding phenotype separation techniques, it is accepted that the immunomagnetic separation of tumour cells using the CD44 marker, which is a marker for many types of cancer stem cells, including breast CSCs, allows lineages with greater tumourigenicity and metastatic potential to be obtained from breast tumours [12], as well as colorectal [16], pancreatic [15], and prostate tumours [18]. In addition, this marker is expressed in carcinoma cell lines, where it plays a role in the migration of cancer cells and matrix adhesion in response to certain cellular microenvironments, thus improving tumour cell aggregation and growth [19], but CD44 is also expressed in mesodermal cells, such as haematopoietic, fibroblastic, and glial cells.

Much remains to be known about the biological differences between the TNBT CSC subpopulations that would allow us to understand their participation in the carcinogenesis of TNBT. The objective of this study was to determine some biological differences not yet well described in a subpopulation of breast CSCs with the CD44^*high*^ and CD24^*low*^ immunophenotype of the MDA-MB 436 and MDA-MB-231 TNBT cell lines in order to obtain expanded and stable subpopulations that produced stable exosomes. These were also homogeneous subpopulations in which we looked for differences in the content of markers and therapeutic targets in the CSCs of TNBTs.

## 2 Material and methods

### 2.1 Cells and culture medium

The cell lines were MDA-MB-436 (ATCC® HTB-130™) and MDA -MB 231 (ATCC® HTB 26™), both cell lines of human TNBT of the transcriptional claudin-low subtype [20] (ATCC. USA). They were maintained and expanded in DMEM-F-12 culture medium (Sigma) supplemented with 4.5% glucose, 4 mM l-glutamine, 1% antibiotics (Merck-Millipore), 1% essential amino acids (MEM, Sigma), and 10% foetal bovine serum. The difference between the cell cultures was that MDA-MB-436 cells were also supplemented with 0.01 mg/ml insulin (Sigma) and 16 mcg/ml glutathione (Sigma).

### 2.2 Immunoselection of cells

The protocol used in this work established two stages of immunomagnetic cell selection using the MiniMACS magnetic separator (Miltenyi Biotec). After reaching 70% cell confluence, the cells were detached with 0.025% trypsin, centrifuged at 300*g*, and concentrated to a total of 10^7^ cells. Then, CD24-biotin and CD44 antibodies labelled with MACS MicroBeads (Miltenyi) were used to label the target cells.

The first stage was negative selection to obtain a cell population with a CD24^-^ immunophenotype using an LD column (Miltenyi). Then, the cells of the CD24^-^ population were subjected to positive selection by being passed through LS columns (Miltenyi) for the magnetic capture of cells with a CD44^+^ phenotype, thus yielding a population of cells with the CD44^+^/CD24^-^ phenotype, which was corroborated by flow cytometry as described below. Cells with this phenotype were seeded in 175-cm^2^ flasks (SPL Life Sciences Co., Ltd., Korea) and expanded in an incubator (Nuve Incubator, EC 160, Turkey) at 37 °C, 5% CO_2_, and 90% humidity before being cryo-frozen at -170 °C in liquid nitrogen.

### 2.3 Characterization of cancer stem cells

#### 2.3.1 Cytometry

To corroborate the selected phenotype and determine the population size that had this phenotype in the cell isolates obtained and expanded, flow cytometry was performed using a Guava Easy-Cyte™ (Merck/Millipore) using CD24-PE (MACS Cod. 130-098-861) and CD44-FITC (Abcam ab95138) monoclonal antibodies in cell cultures of the 2nd-5th passage after thawing.

#### 2.3.2 Determination of immunophenotype by polymerase chain reaction (PCR)

Cells with a CD44^*high*^/CD24^l*ow*^ immunophenotype were expanded in a 100 × 20 mm Petri dish, then detached by a cell scraper, counted, and deposited in a cryovial at a population of 2×10^6^ dissolved in RNAlater (Sigma) for cryopreservation at -70 °C until processing. The applied protocol was based on King (2010) [21]. The primers described in Table 1 were used.

**Table 1:** Primers used for RT-PCR of Oct4 and Nanog immunophenotypes.

Primer name	Sequence
OCT4H-F	GGAGGAAGCTGACAACAATG
OCT4H-R	CAGGCACCTCAGTTTGAATG
NANOG-F	ACTGTCTCTCCTCTTCCTTC
NANOG-R	CCTGATTGTTCCAGGATTGG
GADP HH-F	CATCTTCCAGGAGCGAGAT
GADP HH-R	TTTCTAGACGGCAGGTCAG

#### 2.3.3 Scanning electron microscopy

Scanning electron microscopy (INSPECT S50 SEM. FEI, Hillsboro, Oregon. USA) was done following the protocol described by Miko et al. (2015) [22], using CD44^*high*^/CD24^*low*^ cells that grew on coverslips to 70% confluence.

### 2.4 Cell proliferation

To determine the proliferative capacity of the immunophenotype CD44^*high*^/CD24^*low*^ of the MDA-MB-231 and MDA-MB-436 TNBT cell lines, 30,000 cells per well were plated in triplicate in 24-well plates in standard medium. The number of cells was counted 24, 48, and 72 h after plating using a haemocytometer. The medium was changed every 2 days. The growth curve was drawn so that the horizontal coordinate defined the hours and the vertical coordinate defined the cell density. The cell doubling time during logarithmic growth was calculated according to the following formula [23]:

Doubling time = Length Log (2) / [ Log (Final concentration) - Log (Initial concentration)]

### 2.5 Tumourigenic capacity in mice

We worked with female BALB/c mice from a second calving purchased from the National Institute of Health of Peru (Instituto Nacional de Salud - INS); they remained in the biotherium of the Universidad Científica del Sur (Scientific University of the South) for 15 days before the start of the experiment, fed a diet purchased from INS, water ad libitum and 12 h of light per day. They were kept in groups of 6 animals per cage. This study was conducted in strict accordance with the recommendations of the INS Guide for the Care and Use of Laboratory Animals. The research protocol with animal experimentation was approved by the Scientific Ethics Committee of Universidad Científica del Sur (Resolution No. 005-2013).

Worked was conducted with 48 animals that were distributed in four groups (G-1, G-2, G-3, and G-4), and each group had two subgroups of six animals each. One subgroup received intravenous inoculation of 2×10^6^ cells, and the other subgroup was inoculated subcutaneously (SC) with 2×10^5^ cells. G-1 was inoculated with CD44^*high*^/CD24^*low*^ cells from the MDA-MB-231 line, G-2 was inoculated with CD44^h*igh*^/CD24^*low*^ cells from the MDA-MB-436 line, G-3 was inoculated with the MCF-7 line, and G-4 was inoculated with saline solution.

Previously, all animals had been subjected to a dexamethasone immunosuppression protocol consisting of a first intraperitoneal dose of 200 mg/kg on the first day and 15 days later the second intraperitoneal dose of 30 mg/kg. Immediately, the cells with a CD44^*high*^/CD24^*low*^ immunophenotype were inoculated intravenously. After 30 days of cell inoculation, all animals were sacrificed by intramuscular application of pre-anaesthetic xylazine (10 mg/kg) and then sodium pentobarbital (150 mg/kg) intraperitoneally to detect the presence of tumours and collect samples for histological and immunohistochemical experiments.

### 2.6 In situ immunodetection of xenotransplanted cells

Xenotransplanted cells were detected in paraffinized tissues by immunohistochemistry using a human cell–specific anti-nucleus antibody (Anti-Nuclei Antibody, clone 235-1, MAB1281, Millipore). To this end, sections of submandibular, breast, and lung tumours were fixed in 10% buffered formalin to obtain 5-μm paraffinized histological sections. After deparaffinization, an antigenic recovery protocol was applied using citrate buffer pH 6.0 in a microwave oven (LG) at power level 4 for 5 min. Then the manufacturer protocol of the kit for immunohistochemistry (Invitrogen) using 3,3′-diaminobenzidine as chromogen was followed. To take immunocytochemistry photographs, a NIKON Eclipse T 200 microscope was used, with a Canon camera and software for image processing.

## 3 Results

### 3.1 Magnetic immunoselection

Following the magnetic immunoselection protocol for the CD44 and CD24 surface markers, four immunophenotypes were obtained: CD44^*high*^/CD24^*low*^, CD44^*high*^/CD24^*high*^, CD44^*low*^/CD24^*high*^, and CD44^*low*^/CD24^*low*^, of which the first is the accepted immunophenotype of CSCs of TNBTs. To work with a desirable sample uniformity in terms of the number of passages, immunoselection was performed the same day, and for the selection of the CD44^*high*^ lineage, a population of 5.55×10^6^ cells was used.

Immunoselection of the MDA-MB-231 line (Table 2) produced a population of CSCs with a CD44^*high*^/CD24^*low*^ immunophenotype equivalent to 18.75% of the total p0opulation of this cell line, while the CD44^*high*^/CD24^*high*^ immunophenotype represented 30.25% of the largest subpopulation.

**Table 2:** Immunophenotypes obtained by magnetic selection from the lines MDA-MB-231 and MDA-MB-436 (ATCC).

IMMUNOPHENOTYPE	PERCENT OF CELLS	
	MDA-MB-231	MDA-MB 436
CD44^high^/CD24^low^	18.75	14.56
CD44^high^/CD24^high^	30.25	35.20
CD44^low^/CD24^high^	26.50	25.52
CD44^low^/CD24^low^	23.40	23.19

In the MDA-MB-436 line, the CSCs with a CD44^*high*^/CD24^*low*^ immunophenotype made up 14.56% of the total number of this cell line with which immunoselection was initiated, while the CD44^*high*^/CD24^*high*^ immunophenotype made up 35.20%, being the largest subpopulation (Table 2).

**Figure 1:**
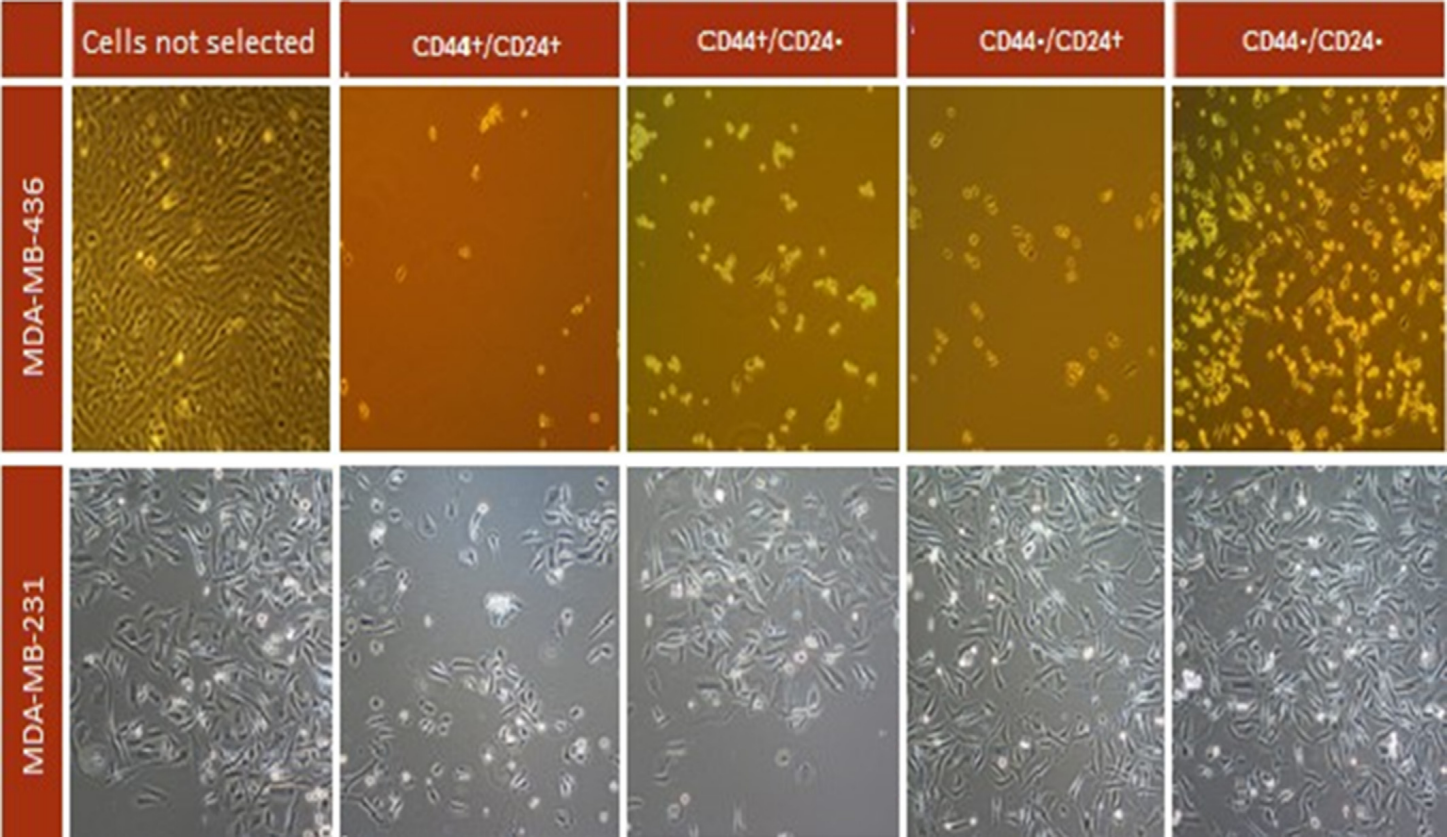
Morphology, seen under inverted microscope, of the different immunophenotypes obtained by magnetic immunoselection of the MDA-MB-231 and MDA-MB-436 lines (48-hour culture).

Figure 1 shows the morphology of the different immunophenotypes of the two cell lines under study at 48 h under an inverted microscope. The CD44^*high*^/CD24^*low*^ phenotype had a moderate proliferation rate, while theCD44^*low*^/CD24^*low*^ phenotype showed a higher proliferation rate (Figure 1).

**Figure 2:**
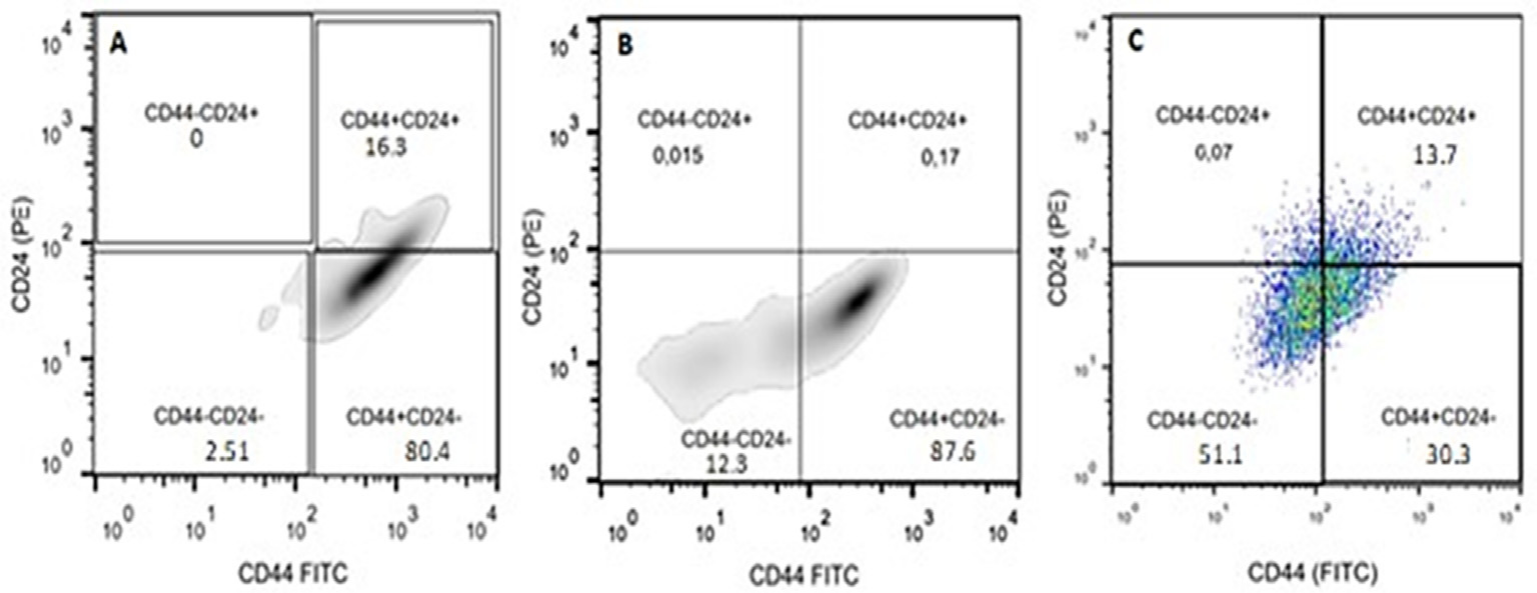
Flow cytometry. (A) MDA-MB-231 cell line, (B) MDA-MB-436 cell line, and (C) MCF-7 cell line.

The CD44^*high*^/CD24^*low*^ immunophenotype corresponding to the CSC of each cell line was expanded in a culture medium similar to that used for immunoselection. The immunophenotypes were expanded until they reached the number of cells required by the immunomagnetic selection protocol applied in this work. Then, by flow cytometry, we verified that the MDA-MB-231 line had an 80.4% purity of this immunophenotype, the MDA-MB-436 line, 87.6% in the 5th passage after thawing**.** For the MCF-7 line, only populations with 30% of the CSC immunophenotype were obtained, with which xenotransplantation in mice was performed (Figure 2).

### 3.2 Determination of immunophenotype by RT-PCR

**Figure 3:**
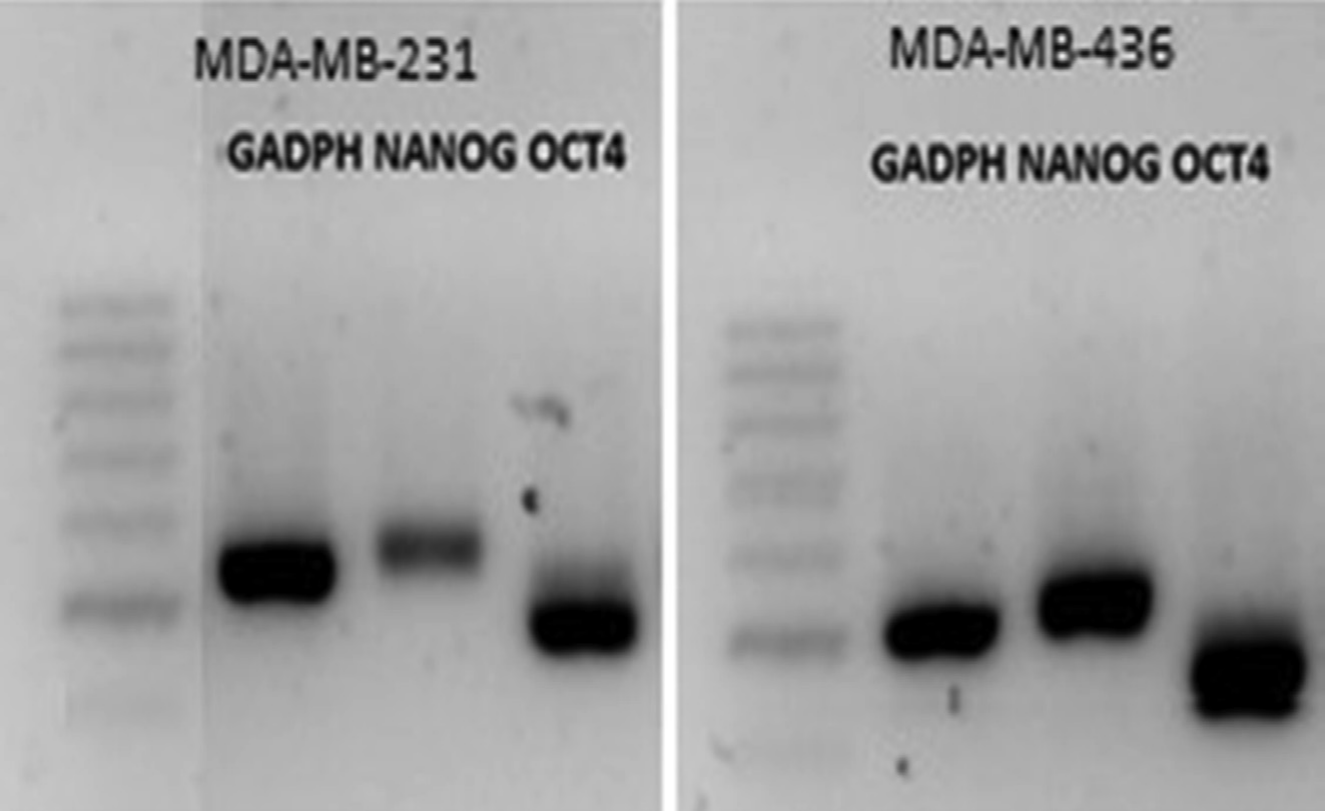
RT-PCR for the pluripotency markers Nanog and Oct4.

Cells of both lines with a CD44^*high*^/CD24^*low*^ immunophenotype were shown to express the pluripotency markers Oct4 and Nanog (Figure 3).

### 3.3 Scanning electron microscopy

**Figure 4:**
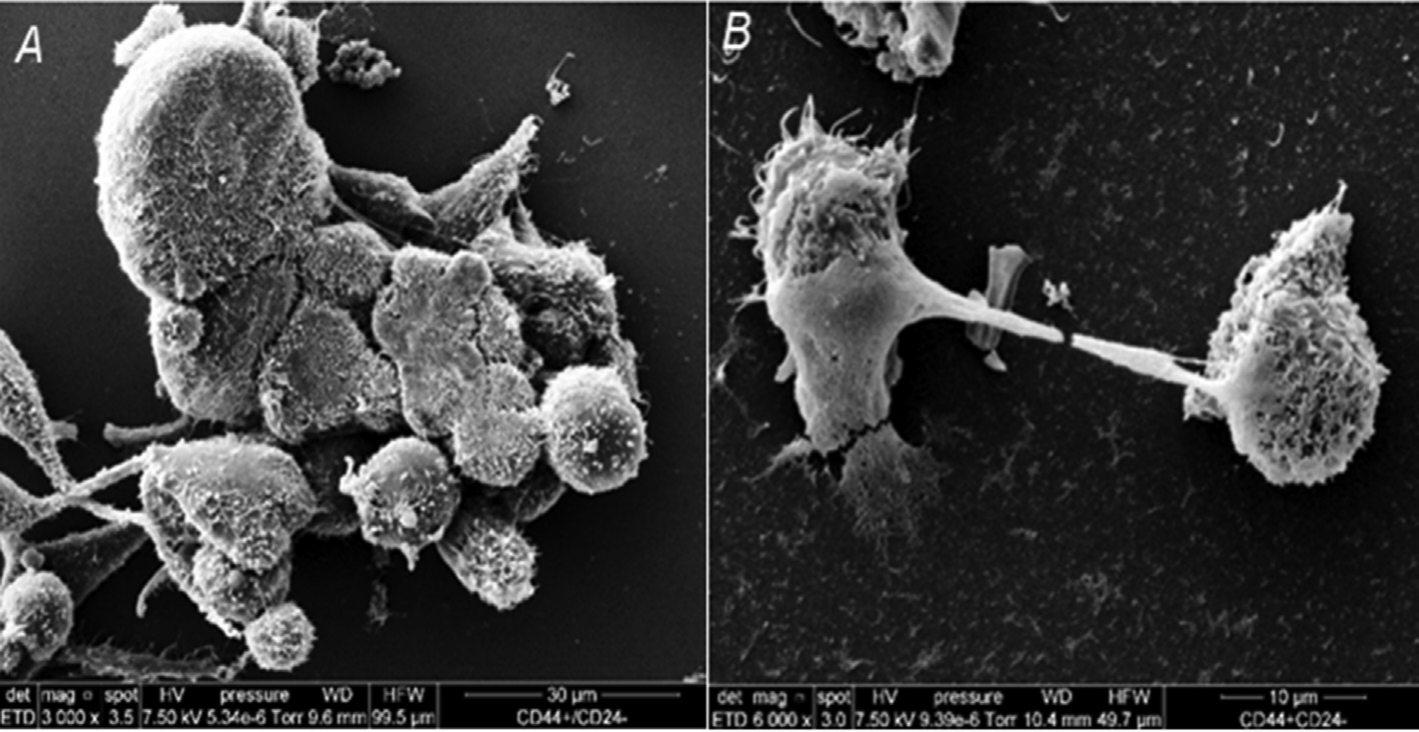
Scanning electron microscopy. (A) MDA-MB-231 cell line. (B) MDA-MB-436 cell line.

Scanning electron microscopy showed that the CD44^high^/CD24^low^ lineage cells of the MDA-MB-436 line form clusters with a higher number of intercommunicating cells with numerous short microvilli and well-defined filopodia than those of the MDA-MB-231 line (Figure 4).

### 3.4 Cell proliferation

**Figure 5:**
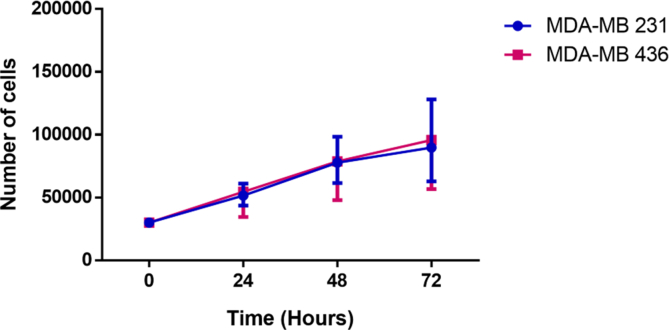
Differences in the cell proliferation capacity of the two cell lines as a function of culture time.

The cell counts performed over 72 h of the CD44^*high*^/CD24^*low*^ immunophenotype of the cell lines studied showed that the MDA-MB-231 line had a lower doubling time (41.56 h) than the MDA-MB-436 line (44.79 h) (Figure 5).

### 3.5 Tumourigenic capacity in mice

**Figure 6:**
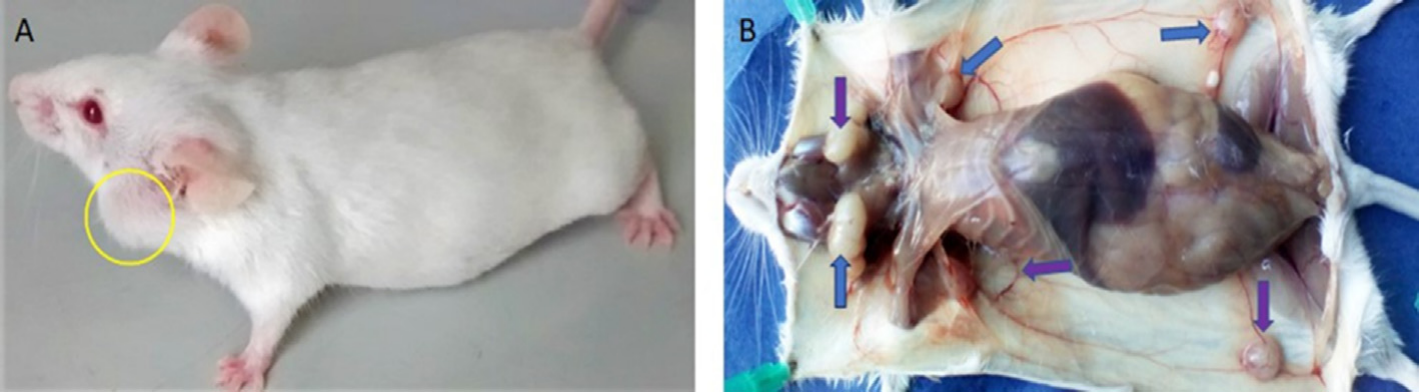
Inoculated mouse with CD44^*high*^/CD24^*low*^ immunophenotype cells of the MDA-MB-231 line, showing development of tumours: (A) Submandibular. (B) Breast (arrows in inguinal region) and submandibular (arrows).

Tumours formed in 16% of either group of immunosuppressed multiparous female mice intravenously (2×10^5^ cells) or subcutaneously xenotransplanted. Tumour development was seen 30 days after inoculation of the cells of both lineages (Figure 6). The non-triple-negative control cells corresponding to human breast tumours of the MCF-7 cell line did not produce tumours in the inoculated animals within the same time.

**Figure 7:**
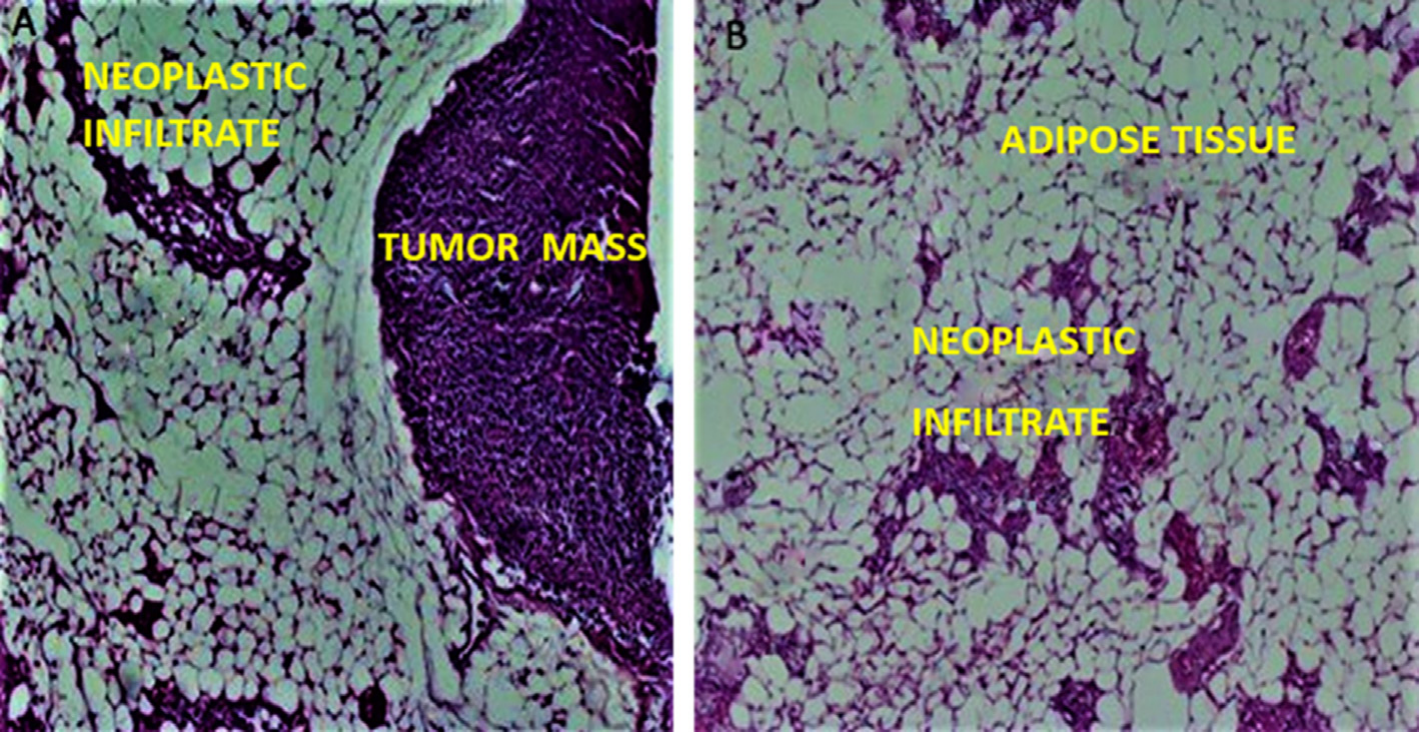
Histology of breast tumours in mice inoculated with MDA-MB-231 cells. (A) Breast tumour. (B) Neoplastic cells infiltrate in breast subcutaneous adipose tissue. (10X, H/E staining.)

The MDA-MB-231 cell line induced the formation of two breast tumours of 0.8 cm (Figure 7-B), as well as infiltration of tumour cells in adipose tissue (Figure 7-A), in addition to submandibular tumours (Figure 6).

**Figure 8:**
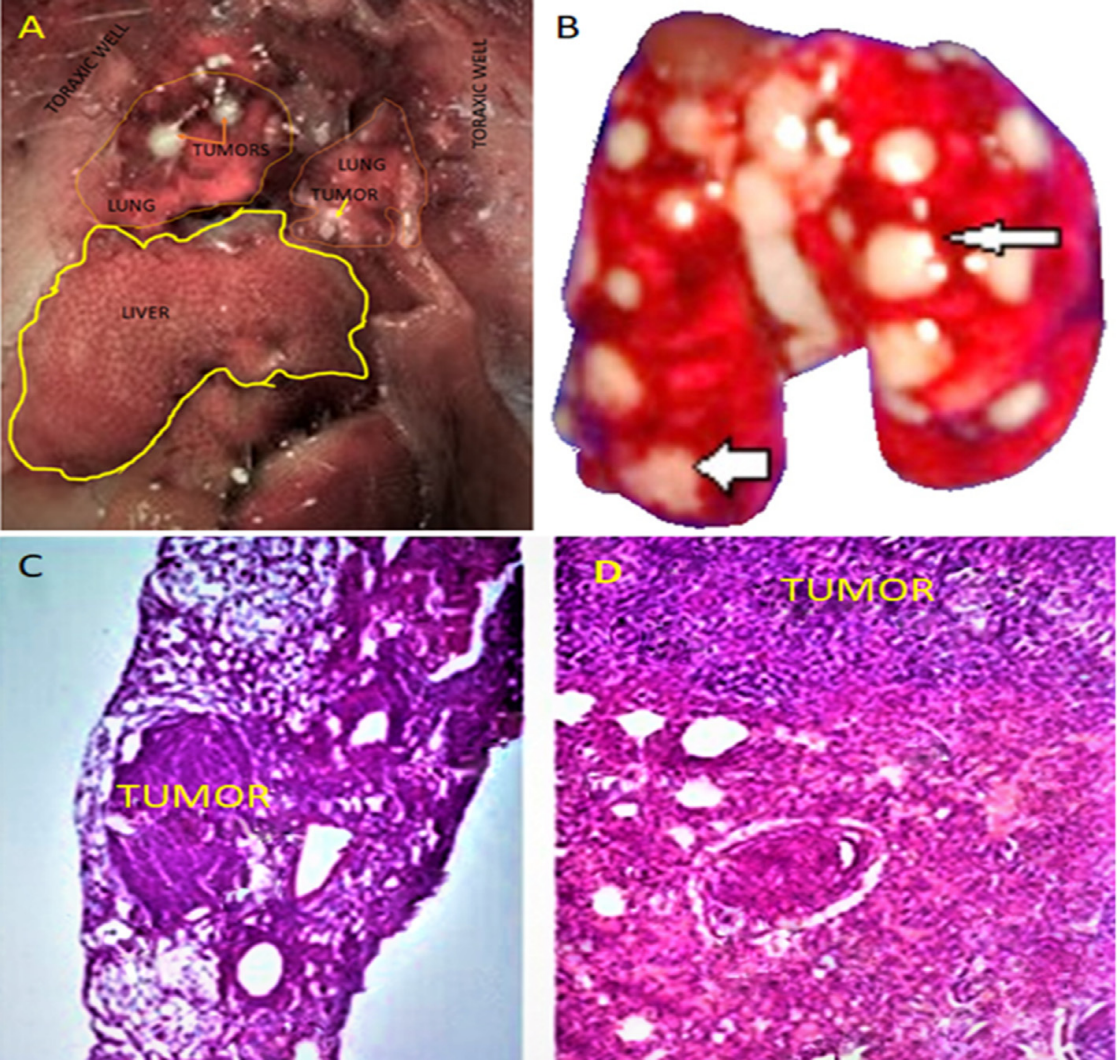
Lung tumours (yellow asterisks) (A) in mice inoculated with CD44^*high*^/CD24^*low*^ cells of the MDA-MB-436 line. Multiple white tumours in the lungs (arrows). (C) Histology of the lung, 10× and (D) 20×. (H/E staining.)

CD44^*high*^/CD24^*low*^ cells of the MDA-MB-436 line inoculated intravenously and subcutaneously into mice immunosuppressed with dexamethasone produced multiple lung tumours after 30 days (Figure 8 A-B). These tumours were histologically described as lung carcinoma (Figure 8 C and D) in 16% of mice.

### 3.6 Immunodetection of xenotransplanted cells

**Figure 9:**
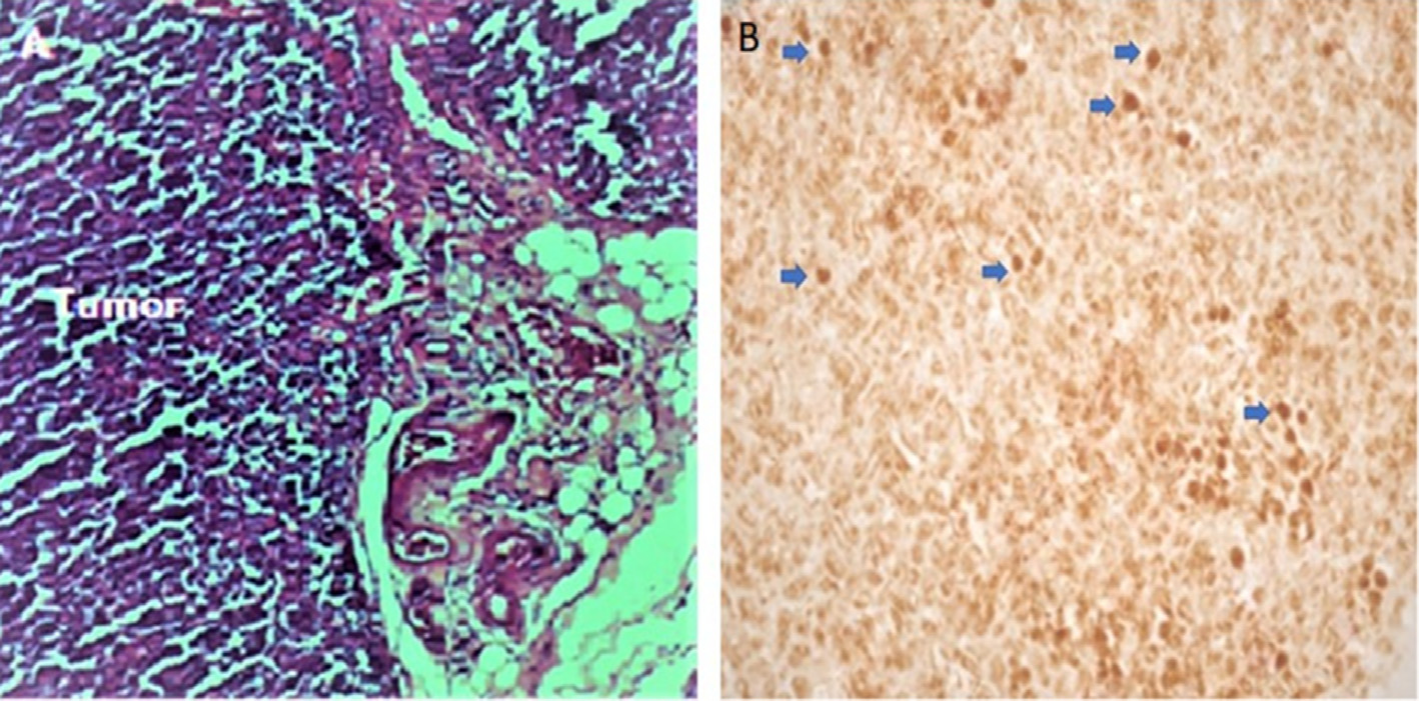
(A) Tumour histology (H/E staining, 10×). (B) Immunodetection of human cells (blue arrows) in tumour tissue of mice inoculated with CD44^*high*^/CD24^*low*^ cells (IHC with immunoperoxidase).

**Figure 10:**
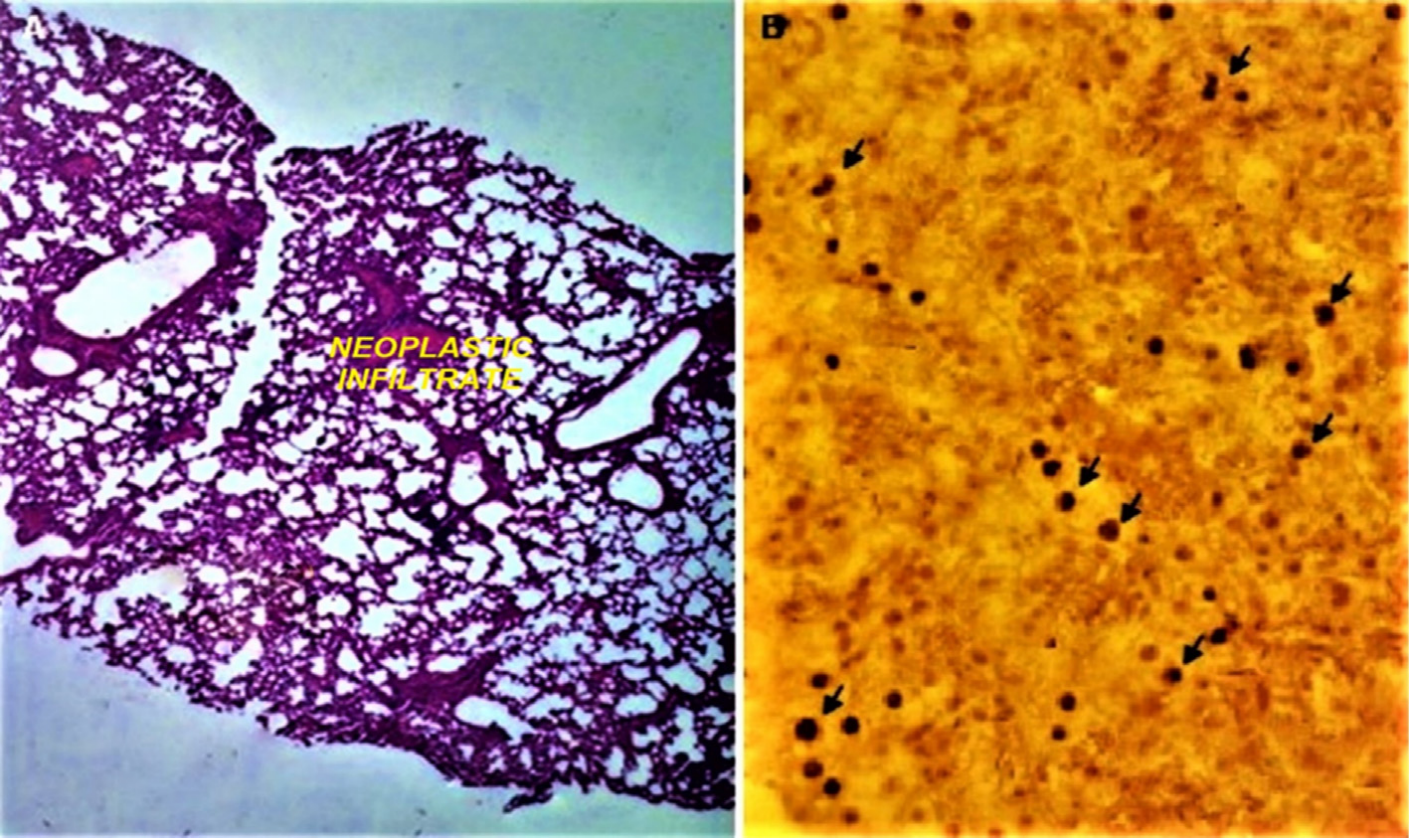
(A) Lung histology (H/E staining, 10×). (B) Immunodetection of human cells (black arrows) in lungs of mice inoculated with CD44^*high*^/CD24^*low*^ cells of the MDA-MB-436 line (IHC with immunoperoxidase).

Cells of human origin were identified by immunohistochemistry using a monoclonal human and nonhuman primate cell–specific anti-nuclear antibody. We observed a pattern of intense brown nuclear staining in the lung and breast tumours of female mice intravenously and subcutaneously inoculated with CD44^*high*^/CD24^*low*^ cells (Figures 9 and 10).

## 4 Discussion

Breast cancer is a very heterogeneous disease whose intrinsic molecular subtypes can explain the intertumoural heterogeneity, while the cancer stem cell hypothesis would explain its intratumoural heterogeneity. In this latter hypothesis, the CD44^*high*^ phenotype is a prominent CSC antigen, while the CD44^*high*^/CD24^*low*^ phenotype is one of the main markers of these cells in invasive breast cancer [24] and of poor prognosis in TNBT [25].

Based on this knowledge, we achieved the isolation and expansion of the CD44^*high*^/CD24^*low*^ immunophenotype of two TNBT cell lines. This immunophenotype is attributed to breast cancer stem cells. For this purpose, we applied the immunomagnetic selection technique using magnetic beads, which after population expansion yielded a purity of 80–90% of the immunophenotype, a result that corroborates the high specificity and sensitivity indicated by other authors for this technique [26]. The isolation and biological study of this subpopulation of tumour cells is deemed important because they are thought to be important in the initiation and recurrence of breast cancer TNBT [27].

After the isolation and expansion of the CSC immunophenotype of the two lines under study, we determined that they differed in their proliferative capacity in the culture medium recommended for each line. The MDA-MB-231 line had better proliferative capacity, showing a lower population doubling time than the MDA-MB-436 line. Some authors attribute differences in population doubling time between breast tumour cell lines to differences in culture time, number of passages [28], and cell plating density [29]. Under scanning electron microscopy, this immunophenotype of the MDA-MB-231 line showed greater intercellular adhesion than the other line, as the cell clusters had a greater number of cells. These dissimilar biological characteristics between the CSCs of the two cell lines could explain the faster proliferation of CD44^*high*^/CD24^*low*^ cells of the MDA-MB-231 line, since the greater intercellular adhesion of this line would allow a greater exchange of growth and related molecules immersed of the secretome, with the consequent stimulation of the cell cycle.

In this increase in cell adhesion, collagen plays an important role, as has been shown in mesenchymal stem cells, which secrete collagen in greater quantities than other cells, stimulating their proliferation [30]. This may suggest that the isolated CSCs in this study secreted a high amount of collagen, thus explaining the rapid proliferation of the CD44^*high*^/CD24^*low*^ MDA-MB-231 cells. If they do, then it will be necessary to study the role of collagen in the future. However, it should also be considered that CSCs have a remarkable ability to reprogram their metabolism [31], and the consequence of this reprogramming may affect their proliferation.

On the other hand, the magnetic immunoselection performed in cell cultures of the two lines allowed four immunophenotypes to be obtained, with the CD44^*high*^/CD24^*low*^ CSCs being those with the lowest population and the CD44^*high*^/CD24^*high*^ those with the highest population. The other immunophenotypes CD44^*low*^/CD24^*high*^ and CD44^*low*^/CD24^*low*^ each represented a quarter of the total population in both cell lines. In addition, morphological differences between all immunophenotypes were shown. Other researchers have shown that certain immunophenotypes are related to resistance to chemotherapeutic agents in breast tumours [17].

However, it is not known precisely how important these population differences are, because although the CD44^*high*^/CD24^*low*^ phenotype is mesenchymal and more invasive than the other epithelial phenotypes, the remarkable plasticity and dynamic expression of the phenotypes of the tumour cells has been shown. Thus, both in vitro and in vivo, a non-invasive phenotype is transformed into an invasive phenotype and vice versa [32]. In future studies of this type of cancer, given the importance of different cellular-phenotype populations, more studies are needed to establish which factors induce these phenotypic changes, as there would be molecules or factors in vivo, such as stromal factors, that would induce dynamic molecular interactions that would lead to changes from the non-invasive cellular phenotype to the invasive phenotype and vice versa [33].

On the other hand, PCR for Oct4 and Nanog was performed on the CD44^*high*^/CD24^*low*^ cells immunoselected in this work. Both lineages expressed both of these pluripotency markers, so we conclude that cells with this phenotype in both cell lines have phenotypic characteristics of CSCs. Subsequently, these isolated cells were expanded for two passages, after which they had reached the population necessary for inoculation in mice. This phenotype was previously shown by flow cytometry to make up 80–90% of the CSC population.

The CSCs with the CD44^*high*^/CD24^*low*^ phenotype isolated and characterized in this work, which originated from two lines taken from pleural effusions, showed invasion and tumourigenic capabilities in female BALB/c mice, although at low percentages, which could be explained by the fact that we worked with immunocompetent mice. However, it is important to note that both lines generated tumours even though the MDA-MB-436 line is designated by the supplier of the line as non-tumourigenic. They could have given rise to tumours because we immunoselected and expanded the CSCs in vitro before xenotransplantation. Thus, our results contrast with the proposal of other researchers who claimed that the CSC lineage is not sufficient for the establishment of lung metastasis [14], while others suggest that this lineage is found in greater quantity in metastatic tumours than in primary tumours or in the parental line [34], which held true of the two parental lines from which the CSCs were obtained in this work.

Although much work has been done with genetically immunosuppressed mice, we decided to work with immunocompetent and immunosuppressed mice treated with corticosteroids because we believe that they better reproduce the natural state of a human patient, allowing us to learn about TNBT carcinogenesis events that more resemble those of the human patient. In addition, because they are tumour cells with CSC characteristics, they have immunosuppressive and immunotolerance effects that would decrease the risk of transplant rejection [35], so we expected a greater percentage success of xenotransplantation of cells with CD44^*high*^/CD24^*low*^ phenotype. However, in this study, we only achieved 16% xenotransplantation in each cell line. This low rate could be attributed to incomplete immunosuppression, low doses, low post-inoculation time, low population doubling capacity, or too many passages of the studied lines.

Cells with a CD44^*high*^/CD24^*low*^ CSC immunophenotype from both lines were found in low quantities in the neoplastic lung parenchyma and in the brain, without generating tumour tissue, but in greater quantities in tumours up to 30 days after inoculation. These findings suggest that CSCs act through paracrine activity, possibly by still-unknown molecules that could be found in their exosomal vesicles that they discharge into the tissue microenvironment where they are established.

## 5 Conclusions

In this study, magnetic immunoselection allowed us to isolate, characterize, expand, and xenotransplant cells with the CD44^*high*^/CD24^*low*^ phenotype of the two TNBT lines studied. Among cells with a CD44^*high*^/CD24^*low*^ phenotype, the MDA-MB-231 cell line has a shorter population doubling time than the MDA-MB-436 cell line. The CD44^*high*^/CD24^*low*^ phenotype of either cell line produced tumours in 16% of the immunocompetent female mice inoculated at the lymph node and lung levels. Few CSCs with a CD44^*high*^/CD24^*low*^ phenotype persisted in tumour or nontumour tissue more than 30 days after inoculation.

## Declarations

### Author contribution statement

Javier Enciso-Benavides, Luis Alfaro, Nancy Rojas, Fernando Riesco: Performed the experiments.

Carlos Castañeda-Altamirano: Analyzed and interpreted the data.

José González-Cabeza, Miluska Castillo: Contributed reagents, materials, analysis tools or data.

Nathaly Enciso: Conceived and designed the experiments; Performed the experiments; Analyzed and interpreted the data; Wrote the paper.

Javier Enciso: Conceived and designed the experiments; Analyzed and interpreted the data; Wrote the paper.

### Funding statement

This work was supported by Proyecto CONCYTEC–Banco Mundial “Mejoramiento y Ampliación de los Servicios del Sistema Nacional de Ciencia Tecnología e Innovación Tecnológica” 8682-PE (104-2018-FONDECYT-BM-IADT-AV).

### Data availability statement

Data included in article/supplementary material/referenced in article.

### Declaration of interests statement

The authors declare no conflict of interest.

### Additional information

No additional information is available for this paper.
